# Analysis of herbivore-responsive long noncoding ribonucleic acids reveals a subset of small peptide-coding transcripts in *Nicotiana tabacum*

**DOI:** 10.3389/fpls.2022.971400

**Published:** 2022-09-23

**Authors:** Jingjing Jin, Lijun Meng, Kai Chen, Yalong Xu, Peng Lu, Zhaowu Li, Jiemeng Tao, Zefeng Li, Chen Wang, Xiaonian Yang, Shizhou Yu, Zhixiao Yang, Linggai Cao, Peijian Cao

**Affiliations:** ^1^China Tobacco Gene Research Center, Zhengzhou Tobacco Research Institute of CNTC, Zhengzhou, China; ^2^China Tobacco Hunan Industrial Co., Ltd., Changsha, China; ^3^Molecular Genetics Key Laboratory of China Tobacco, Guizhou Academy of Tobacco Science, Guiyang, China

**Keywords:** lncRNAs, small ORF-encoded peptide, herbivore defense, mass spectrometry, tobacco

## Abstract

Long non-coding RNAs (lncRNAs) regulate many biological processes in plants, including defense against pathogens and herbivores. Recently, many small ORFs embedded in lncRNAs have been identified to encode biologically functional peptides (small ORF-encoded peptides [SEPs]) in many species. However, it is unknown whether lncRNAs mediate defense against herbivore attack and whether there are novel functional SEPs for these lncRNAs. By sequencing *Spodoptera litura*-treated leaves at six time-points in *Nicotiana tabacum*, 22,436 lncRNAs were identified, of which 787 were differentially expressed. Using a comprehensive mass spectrometry (MS) pipeline, 302 novel SEPs derived from 115 tobacco lncRNAs were identified. Moreover, 61 SEPs showed differential expression after *S. litura* attack. Importantly, several of these peptides were characterized through 3D structure prediction, subcellular localization validation by laser confocal microscopy, and western blotting. Subsequent bioinformatic analysis revealed some specific chemical and physical properties of these novel SEPs, which probably represent the largest number of SEPs identified in plants to date. Our study not only identifies potential lncRNA regulators of plant response to herbivore attack but also serves as a valuable resource for the functional characterization of SEP-encoding lncRNAs.

## Introduction

Long non-coding RNAs (lncRNAs) are generally longer than 200 nucleotides and lack coding potential (Jin et al., 2013). Commonly, coding potential is rather arbitrary and is generally used to distinguish noncoding RNAs from protein-coding genes according to some features, including open reading frame (ORF) length and sequence similarity. Many algorithms and tools have been developed to classify lncRNAs, including CPC (Kong et al., 2007), PLEK (Li et al., 2014), RNAplonc (Negri et al., 2019), and CPAT (Wang et al., 2013). Although lncRNAs are initially described as mRNA-like transcripts not encoding proteins, many small ORFs (smORFs) embedded in lncRNAs are found to encode functional peptides (smORF-encoded peptides [SEPs]) (Choi et al., 2019). These SEPs may play important roles in various biological processes in humans (D’Lima et al., 2017; Huang et al., 2017; Zhang et al., 2017), other animals (Kondo et al., 2007; Pauli et al., 2014; Nelson et al., 2016), and plants (Rohrig et al., 2002; Lin et al., 2020). For example, functional studies of some well-known mammalian lncRNAs, including *SPAR* (Matsumoto et al., 2017), *HOXB-AS3* (Huang et al., 2017), *DWORF* (Nelson et al., 2016) and *Toddler* (Pauli et al., 2014), have confirmed that some lncRNAs might indeed encode short peptides with key biological functions. A study on soybean demonstrated that lncRNA *ENOD40* might encode two short peptides required for plant–bacteria symbiotic interactions by interacting with sucrose synthase genes (Rohrig et al., 2002). Considering such large number of smORFs in lncRNAs, majority of SEPs have not been annotated yet. Hence, accelerating the functional identification of unknown SEPs would provide more evidence for exploration of noncoding region in the genome.

As rapid development of high-throughput sequencing technology, thousands of lncRNAs with smORFs have been discovered. Deep sequencing of ribosome-protected mRNA fragments, also known as Ribo-Seq (ribosome sequencing), has revealed that some lncRNAs are strongly associated with ribosomes; however, these associations do not always imply that such lncRNAs are actively translated (Ingolia et al., 2012; Guttman et al., 2013; Ruiz-Orera et al., 2014; Raj et al., 2016; Wang et al., 2017). Furthermore, several studies have attempted to detect peptides encoded by lncRNA transcripts using mass spectrometry (MS; Koch et al., 2014; Sun et al., 2014; Crappe et al., 2015), which ionizes peptides and identifies the corresponding amino aicd (aa) sequence by measuring mass-to-charge ratio. These progress has expanded our understanding of lncRNAs and coding ability of the whole genome.

In nature, plants are frequently suffered by herbivores, most of which are insects. In order to successfully survive during herbivore attack, plants have evolved various physiological and molecular mechanisms. Generally, the herbivore response of plants can be summarized as the following mechanisms: the activation of mitogen-activated protein kinases (MAPK), increased cytosolic calcium levels, and the depolarization of plasma membrane potential (Mostafa et al., 2022). Afterward, phytohormone signals, including jasmonic acid (JA), ethylene, and salicylic acid (SA) are quickly activated (Mostafa et al., 2022). Among them, JA and its bioactive derivatives, collectively known as JAs, have been proven to play a central role in plant defense against herbivores (Wasternack and Hause, 2013; Howe et al., 2018). In addition to core JA pathway, other regulators have also been identified, which may be involved in JA-mediated plant herbivore response. For example, several WRKY transcription factors could act as specific herbivore-induced factors by positively regulating JA upon herbivore attack (Li et al., 2015). Silencing of glutamate receptor-like proteins could reduce JA signal level, and decrease plant defense against herbivores (Toyota et al., 2018). These discovery indicated that plant defense against herbivore was a complex regulatory network, and many other pathways and regulators still need to be elucidated.

Many functional studies have revealed that lncRNAs could involve in various plant development and stress response, including flowering, seed germination, salt, cold and pathogens response (Heo and Sung, 2011; Henriques et al., 2017; Seo et al., 2017, 2019; Wu et al., 2018; Jin et al., 2021). Previous studies have found that small noncoding RNAs might involve in herbivore resistance (Pandey et al., 2008). However, little is known about the role of lncRNAs in JA signaling and herbivore defense (Jin et al., 2021; Li et al., 2021). Moreover, the detailed mechanism for these herbivore response lncRNAs has been rarely explored. Here, using common tobacco, *Nicotiana tabacum*, one of well-known model plants to study its interactions with herbivores, we aimed to identify lncRNAs in tobacco by sequencing *Spodoptera litura*-treated leaves at six time-points over 24 h. Spatiotemporal expression pattern was observed for herbivore response genes. Many lncRNAs were found to response for *S. litura* attack, and might play roles together with JA signals. Using our proposed SEP enrichment workflow, we showed that a portion of the herbivore-response-related lncRNAs might encode small peptides, as supported by MS evidence. Subsequently, we tried to verify the existence of identified SEPs by multiple technologies. This study not only revealed the new role of lncRNA during herbivore response, but also revealed the true identity of such SEPs as small peptides, rather than as lncRNAs.

## Materials and methods

### Plant materials and sample collections

*Nicotiana tabacum* germplasm K326 was used in this study. Control plants were grown under normal conditions, as previously described (Jin et al., 2017; Li et al., 2021).

*Spodoptera litura* specimens were obtained from in-house colonies. Then, we collected oral secretions (OS) from 4th-instar larvae. Similar to our previous study (Jin et al., 2021; Li et al., 2021), for *S. litura* treatments, leaves from the sixth leaf-growth stage were wounded using a pattern wheel. Then, OS (20 μl) was rubbed into the stab. For each treatment, five to eight plants were used. Untreated plants were regarded as controls.

### Hormone analysis

Samples of leaf tissues were ground and extracted with ethyl acetate (800 μl) containing the internal standards. All control and treated samples were analyzed by UHPLC-HESI-MS/MS as previously described (Li et al., 2021).

### Annotation of long non-coding RNAs

Strand-specific RNA sequencing was conducted by Novogene Company. Clean data were aligned to the reference tobacco genome (Edwards et al., 2017) by HIAST2 with parameter – rna-strandness RF (Kim et al., 2015). The transcriptome dataset for each library was separately assembled by StringTie (Pertea et al., 2015). Then, gtf files for each library were combined into one with StringTie–merge. The assembled transcripts were compared with reference genome annotation. Transcripts with a length shorter than 200nt were discarded. The transcripts were further filtered if they were overlapping with tRNA, rRNA, sRNA, and miRNA in Rfam (Mistry et al., 2021) database. Coding potential of the remaining transcripts were measured by the CPC (Kong et al., 2007) programs. The left transcripts were regarded as lincRNA candidates if they located in intergenic regions. Transcripts located in intron region of known genes were considered as incRNA candidates. If transcripts were transcribed from the opposite strands of known genes, they were identified as NATs (natural antisense transcripts) candidates. These transcripts were also compared with lincRNA and NATs annotation from our previous studies in PLncDB (Jin et al., 2021). The expression values for lncRNAs were measured using TPM as previously described (Jin et al., 2021). By comparing control and treated samples at each time point, differentially expressed lncRNAs were defined with absolute fold change (FC) ≥ 1.5 and *p* < 0.05.

### Correlation and enrichment analysis

The correlation between differentially expressed genes (DEGs; FC ≥ 2 and *p* < 0.05) and lncRNAs was determined using weighted correlation network analysis (WGCNA; Langfelder and Horvath, 2008) with default parameters. The interaction network was visualized by Gephi software (Bastian et al., 2009).

Gene Ontology (GO) enrichment analyses of corresponding genes were conducted using KOBAS (Bu et al., 2021). GO terms with a corrected *p*-value smaller than 0.05 were regarded as significantly enriched terms. Gene networks analyses were performed using the NetworkAnalyst software (Zhou et al., 2019), using confidence score with cutoff 900 and experimental evidence required. Furthermore, GO enrichment was performed for each identified sub-network with default parameters.

### Construction of a putative small ORF-encoded peptide database

ORFfinder was applied to lncRNA transcripts to ensure that we could cover all possible smORFs. This resulted in 817,862 polypeptides, which were regarded as putative SEPs. SEPs with lengths of 5–100 amino acids will be considered as SEP database for tobacco.

### LC-MS analysis and identification of annotated proteins/small ORF-encoded peptides

Peptide identification and liquid chromatography-tandem mass spectrometry (LC-MS/MS) analysis were performed as previously described (Zhou et al., 2021) for leaf tissues of control and OS treated plants at 24 h. The LC-MS/MS data were analyzed using MaxQuant search (version 1.6.5; Cox and Mann, 2008). In our study, we used two different types of protein database. These two different databases were constructed as follows: (1) Tobacco canonical protein database, obtained from the SOL consisting of 69,500 entries (Fernandez-Pozo et al., 2015); (2) Putative tobacco SEP database for lncRNAs, consisting of 817,862 entries.

To identify novel candidate peptides, data were searched against our above combined database. The parameter setting was as follows: (1) The digestion mode was set to unspecific; (2) Minimum peptide search was set to 5, and maximum peptide search was 30; (3) The variable modifications was set to oxidation (M) and Acetyl (Protein N-term), and fixed modification was set as carbamidomethyl (C); (4) Matches between runs and LFQ options were selected. Other parameters were set to default values.

### Western-blot analyses

Western blotting analysis was conducted based on standard protocols. The antibodies used in our work were as follows: anti-GFP (D153-8) were customized by MBL International.

### 3D structure predictions

The three-dimensional (3D) structure of identified SEPs were predicted by AlphaFold2 software (Jumper et al., 2021). The visualization was conducted by PyMOL software.^1^ The accuracy of the predicted structures was measured on the SAVES server^2^ using ERRAT score.

## Results

### Temporal regulation of gene modules upon *Spodoptera litura* attack

**FIGURE 1:**
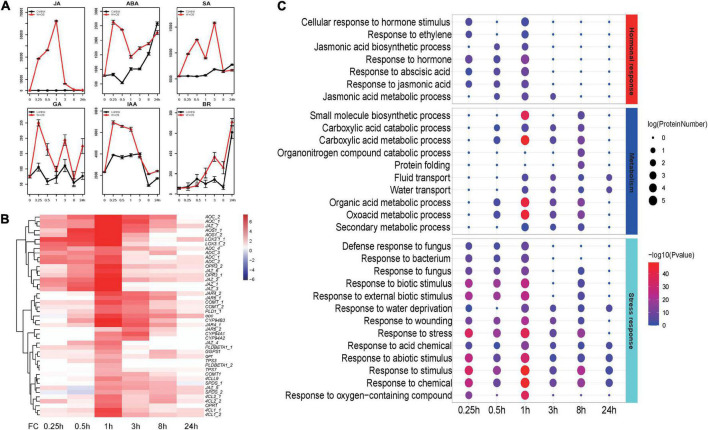
JA and other hormone signals and enrichment analysis for differentially expressed genes in *Nicotiana tabacum* after *Spodoptera litura* elicitation. **(A)** Mean concentrations (Relative Content) of jasmonic acid (JA), abscisic acid (ABA), gibberellic acid (GA), salicylic Acid (SA), indoleacetic acid (IAA), and brassinosteroid (BR) in treated and control leaves. **(B)** Hierarchical cluster analysis of JA biosynthesis and responsive genes. FC: fold change; X-axis represents fold change between treated and control samples. **(C)** GO enrichment results for different time-points based on up-regulated differentially expressed genes.

To analyze temporal responses upon herbivore attack in *N. tabacum*, one of major herbivores, *S. litura* was selected as representative model in our study. Leaves were treated by wounding and then treated with *S. litura* larval oral secretion (OS) at six time-points (0, 0.25, 0.5, 1, 3, 8, and 24 h). Principal component analysis (PCA) analysis revealed that the control and treated samples at each time-point were well separated (Supplementary Figure 1). Compared to control leaves, JAs were significantly increased as early as 15 min after larval OS treatment, and the levels peaked at 1 h after treatment (Figure 1A). Further, compared with JAs, the responses of other hormone signals (ABA: Abscisic acid, GA: Gibberellic acid, SA: Salicylic acid, IAA: Indole-3-acetic acid, BR: Brassinosteroid) were much weaker (Figure 1A). Hence, as the main regulators of plant defense responses against herbivore attack, JAs’ level and JA biosynthesis-related genes were regarded as indicators in the following analysis. Similar to our previous findings in *N. attenuate* (Li et al., 2021), the transcripts of JA-biosynthesis genes (including *JAZs* and JA-related TF genes) accumulated early (<1 h), whereas the effector genes (including terpenoid and phenylpropanoid biosynthesis genes) accumulated later (>3 h; Figure 1B). DEGs were identified at each time-point. Upregulated genes was much larger than that of downregulated genes at all sampling time-points (Supplementary Figure 2A). Overlaps of upregulated DEGs between different time showed a large number (1,682) of genes only upregulated at 1 h (Supplementary Figure 2B). All upregulated DEGs detected were used to explore the enriched biological processes in tobacco triggered during *S. litura* attack. Comparing enriched biological process terms among different time-points, we found that many hormone- and stress-related terms responded early (<1 h), whereas many secondary metabolism terms responded late (>1 h; Figure 1C).

To further investigate the similarities and differences of specific gene modules, networks for upregulated genes were analyzed using NetworkAnalyst (Zhou et al., 2019; Supplementary Figure 2). A time-associated pattern emerged from these networks. Gene modules related to jasmonate-mediated defense responses and signal transduction/perception were rapidly changed before 1 h after attack, as were modules of genes related to hormonal responses associated with growth and defense (Supplementary Figure 3). From 3 h after attack, gene modules related to secondary metabolites biosynthesis, including flavonoids, and terpenoids, were identified (Supplementary Figure 3). Hence, both enriched GO and network module analyses revealed that genes showed a temporal pattern upon *S. litura* attack in tobacco.

### A large number of long non-coding RNAs may be involved in plant responses to *Spodoptera litura* attack

**FIGURE 2:**
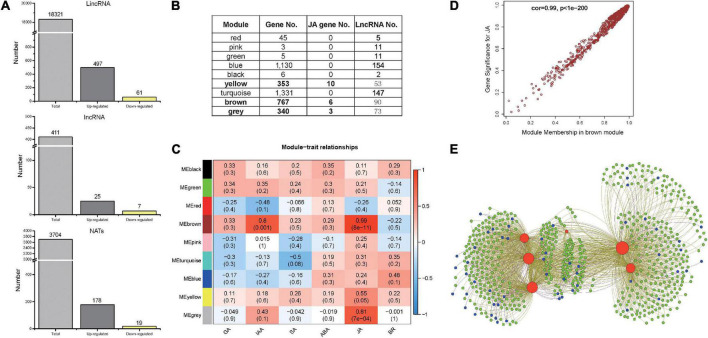
Long non-coding RNAs in *Nicotiana tabacum* after *Spodoptera litura* elicitation and WGCNA analysis for differentially expressed lncRNAs and genes. **(A)** The numbers of identified lncRNAs in *N. tabacum*. **(B)** Each module identified by WGCNA based on differentially expressed genes and lncRNAs. **(C)** The results for module-trait analysis between different module and different hormone signals. **(D)** Gene significance plot between genes in brown module and JA signal. **(E)** Network plot between genes in JA biosynthesis pathways and other genes/lncRNAs in brown module. Red color represents JA biosynthesis genes; Green color represents other protein-coding genes; Blue color represents lncRNAs.

To examine whether lncRNAs participate in the plant response to herbivore attack, approximately 253G RNA-seq reads were used to assemble new transcripts (Supplementary Table 1 and Supplementary Figure 4). Together with annotated genes and previously identified lncRNAs in the PLncDB (Jin et al., 2021), a total of 22,436 lncRNAs were identified, of which lincRNA was the most abundant (81.66%; Figure 2A). Comparing between control and treated samples at each sampling time-point, 787 lncRNAs were differentially expressed during *S. litura* attack (Fold change, FC ≥ 1.5; *p* < 0.05). Among them, 558 lincRNAs showed differential expression, of which 497 were upregulated. Two random candidates were verified by qRT-PCR (Supplementary Figure 5). Time-course expression analysis also indicated that the upregulated lincRNAs could play roles as both early and late regulators during herbivore attack (Supplementary Figure 6). LincRNAs might regulate its neighboring genes by interacting with chromatin proteins. The neighboring genes of upregulated lincRNAs (within 100 kb) were identified, and average correlation coefficient between them was 0.42. Furthermore, GO enrichment analysis suggested that these neighboring genes were enriched in stress related processes and the biosynthesis of secondary metabolites (Supplementary Figure 7).

To identify the main lncRNA regulators of the plant response to herbivore attack, the regulatory relationship between differentially expressed lncRNAs and differentially expressed protein-coding genes (3,980) was subsequently investigated by WGCNA. The co-expression network was divided into nine modules (Figure 2B and Supplementary Figure 8) that consisted different proportions of lncRNAs, ranging from 2 in module MEred to 154 in module MEturquoise. Nineteen well-known JA biosynthesis-related genes were present in three modules, including MEyellow, MEbrown, and MEgrey (Figure 2B). Furthermore, an association analysis between different modules and hormone levels was conducted using WGCNA. The MEyellow, MEbrown, and MEgrey modules were significantly correlated with JA signals (Figure 2C), of which MEbrown module was the most significant. The correlation results showed that protein-coding genes and lncRNAs in these three modules had patterns consistent with JA levels (Figure 2D and Supplementary Figures 9A,C). Furthermore, many lncRNAs in these three modules may have a strong relationship with well-known JA biosynthesis-related genes (Figure 2E and Supplementary Figures 9B,D). Hence, numerous lncRNAs in these three modules were co-expressed with JA biosynthesis-related genes (Figures 2D,E and Supplementary Figure 9), indicating their potential role in herbivore-elicited JA signaling.

### Mass spectrometry evidence for long non-coding RNA-encoded small ORF-encoded peptides

**FIGURE 3:**
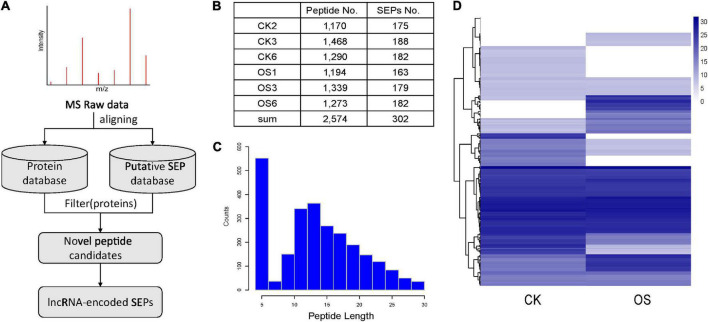
MS-based discovery of lncRNA-encoded SEPs. **(A)** Pipeline for MS-based identification for novel SEPs identification. **(B)** Number of identified peptides and SEPs based on MS data. **(C)** The length distribution of identified peptides. **(D)** Heatmap for the identified SEPs in treated (OS) leaves and control (CK) leaves.

LC-MS/MS data were used to validate the translational profile of tobacco lncRNAs based on the putative tobacco SEP database we constructed (Figure 3A). The proteins were obtained from the leaf tissues of control and OS-treated plants, and fractionated according to hydrophobicity, resulting in 2,574 small peptides (Figure 3B), with lengths ranging from 5 to 30 (Figure 3C). The correlation coefficient between the three biological replicates was very high (Supplementary Figure 10). In order to identify high-confidence lncRNA-encoded SEPs, SEPs detected by MS in our study were strictly filtered (Figure 3A). Peptides would be filtered, if they satisfied any of the following criteria: (1) peptides matched to the annotated tobacco proteins; (2) Length of peptides was less than five. The remaining candidates were regarded as novel peptides derived from SEPs. Totally, 302 SEPs were identified from six tobacco leaf tissues (Figure 3B). As far as we know, this is the largest number of plant SEPs detected by MS. Blast these identified SEPs against the plant small peptide database (PlantProDB; Das et al., 2020) revealed that none of them matched the existing sequences well and were therefore, potentially novel small peptides. Among the identified SEPs, 61 SEPs were detected at least two times in *S. litura* attacked samples, compared to the controls (Figure 3D), implying that some herbivore attack response-related lncRNAs might play their roles as small peptides.

### Validation of the identified novel small ORF-encoded peptides

In order to verify the reliability of the identified SEPs, both bioinformatics techniques (subcellular localization prediction, expression comparison for SEP-encoding lncRNA transcripts and 3D structure prediction) and experimental methods (SEP-encoding lncRNA localization and western blotting) were conducted.

**FIGURE 4:**
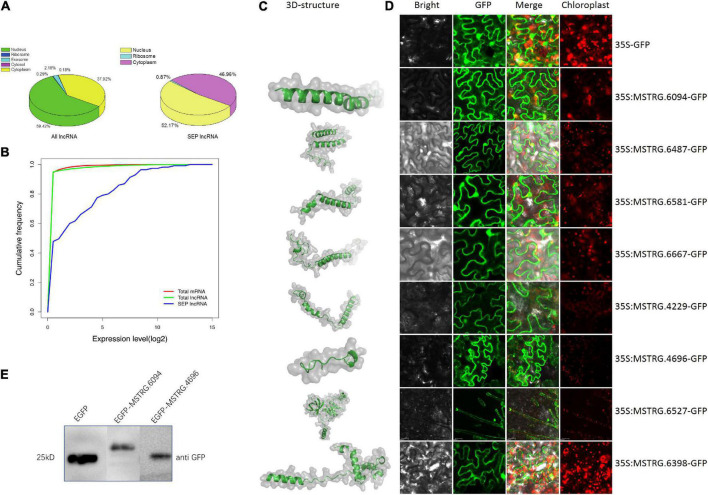
Validation of lncRNA-encoded SEPs. **(A)** Subcellular localization prediction for all lncRNAs and SEP-encoding lncRNAs. **(B)** Expression level comparison between all mRNAs, all lncRNAs and SEP-encoding lncRNAs by cumulative frequency. **(C)** Predicted 3D structure for selected SEPs. **(D)** Subcellular localization of some of SEP-encoding lncRNAs. Scale bars: 20 μm. **(E)** Western blotting results for two SEP-EGFP fusion proteins.

Subcellular localization results using lncLocator (Lin et al., 2021) showed that approximately 50% of SEP-encoding lncRNAs might locate in the cytoplasm, whereas only 38% of the total lncRNAs might locate in the cytoplasm (Figure 4A), indicating that SEP-encoding lncRNAs are more likely to activate translation by binding to ribosomes. The expression levels of RNA transcripts showed that SEP-encoding lncRNAs were slightly higher expressed than total mRNAs and lncRNAs (Figure 4B). The relative higher expression level for SEP-encoding lncRNAs observed here might explain why these novel SEPs were easily discovered in our analysis. To explore the mechanism and function of the small peptides, 3D structures of eleven randomly selected SEPs were predicted using AlphaFold2 (Jumper et al., 2021; Figure 4C and Supplementary Figure 11). Except for one candidate, the ERRAT scores for all predicted 3D models were greater than 80 (Supplementary Table 2), which showed that the predicted 3D structures were reliable.

Furthermore, transient expression experiments in *Nicotiana benthamiana* leaves suggested the plasma membrane localization for most candidates, which implies the ability of ribosomal translation for these candidates (Figure 4D). Interestingly, one SEP-encoding lncRNA (MSTRG.6527) was located in the trichome tissue, suggesting its important function in defense against herbivore attack (Figure 4D). Western-blot result further confirmed two of these candidates (Figure 4E).

Altogether, our analyses verified the reliability of multiple SEPs using different techniques, such as subcellular localization validation, lncRNA expression, MS, and western blotting.

### Characteristics of identified small ORF-encoded peptides

In order to deeply understand the characteristics of identified SEPs, we explored them from several perspectives, such as type by location, length comparison, amino acid usage, start codon usage, and various protein characteristics.

**FIGURE 5:**
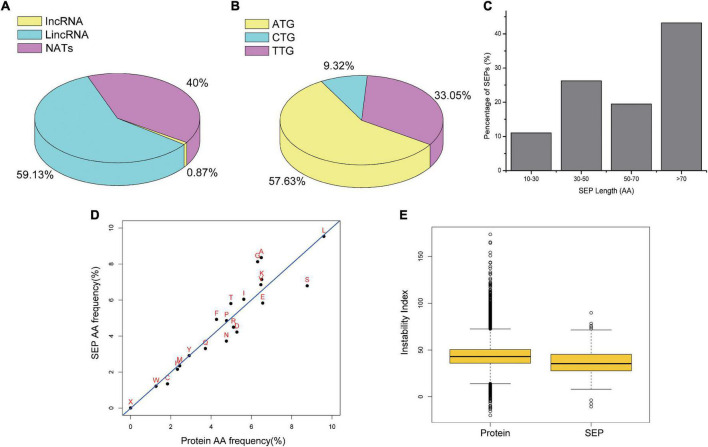
Characteristics of Identified SEPs. **(A)** Pie chart for different class of SEP-encoding lncRNAs. **(B)** Par chart for start codon of identified SEPs. **(C)** Histogram for length of identified SEPs. **(D)** Scatter plot between amino acid usages of proteins and identified SEPs. **(E)** Bar plot for instability index between canonical proteins and SEPs.

According to the genomic location, lncRNAs can be divided into lincRNAs, incRNA, and NATs. Compared with incRNAs and NATs, lincRNAs have a much similar pattern with mRNAs in transcriptional activation mode (Ma et al., 2013). Similarly, among the total identified tobacco SEPs, we also found that SEPs encoded by lincRNAs accounted for 59.13%, whereas NATs and incRNAs accounted for only 40 and 0.87%, respectively (Figure 5A).

Commonly, codon usage preference is one of important features of genome evolution, which provides important clues for the study of gene function. Start codon for identified SEPs was used to analysis codon usage bias in our study. Among the identified tobacco SEPs, 57.63% were activated with an ATG start codon or a nearby cognate start codon (TTG, 33.05%; CTG, 9.32%; Figure 5B). The fact that most SEPs are initiated with a known start codon also implies their coding potential.

We determined the length of lncRNA-encoded SEPs using the predicted ORF length. Most of the identified tobacco SEPs (Figure 5C) had a length in the region of 30–50 and 70–100 aa, and the shortest length was only 13 aa. Further, as nearly 90% of all identified SEPs were longer than 30 aa, enzyme-based digestion MS method may be a good choice for identifying SEP-encoding lncRNAs.

Although we could not totally explore protein function just using its aa sequence, protein sequence could still reflect protein structure and function to some extent. Sequence analysis revealed that identified tobacco SEPs tended to use more positively charged aa (e.g., K) and fewer negatively charged aa (e.g., E and D) than proteins. However, uncharged amino acids usage was similar (Figure 5D), which is consistent with previous study (Lu et al., 2019).

Various protein indices, including protein instability index, molecular weight, isoelectric point, and aromaticity, were characterized by ExPASy ProtParam. No significant differences between the identified SEPs and proteins were found for instability index, aromaticity, and fraction of the helix (Figure 5E and Supplementary Figure 12), in agreement with previous report (Zhang et al., 2021). Differences regarding other protein features, including molecular weight, isoelectric point, and cysteine/disulfide bridges, may be due to the difference in length. These results suggest that our identified SEPs may be as stable as proteins.

## Discussion

As development of next-generation sequencing technology, it was possible to widely explore the functions of lncRNAs in plants (Jin et al., 2021). Defense-related lncRNAs were recently considered as potential regulators of plant-bacteria, plant-fungus, and plant-virus interactions (Seo et al., 2017, 2019; Jin et al., 2021; Li et al., 2021). By sequencing six time-point samples under *S. litura* treatment, 22,436 lncRNAs were identified in the tobacco cultivar used. Among these, 787 lncRNAs were responsive to *S. litura* attack. WGCNA analysis revealed that the MEbrown module was highly correlated with JA levels, indicating a potential role for lncRNAs in herbivore-elicited JA signaling. However, the detailed mechanisms of these specific candidates require further exploration.

Technological advances have accelerated the discovery of large number of important SEPs in many species. However, owing to small size and relatively low abundance, many more are expected to be discovered yet. Hence, sample preparation should be carefully considered and a comprehensive reference database should be built. To construct a reference database which could contain all SEPs in tobacco, transcripts of lncRNA were scanned using ORFinder to enable the representation of putative SEPs (Figure 3A), which resulted in 817,862 polypeptides in our constructed putative tobacco SEP database. Based on our SEP identification pipeline, we finally discovered 302 SEPs from the leaf tissues of control and OS-treated tobacco plants. As far as we know, this is the maximum number of SEPs detected using MS in plants ever to be reported (Das et al., 2020). The improved SEP discovery rate is due to the following reasons. First, our constructed putative SEP database contained the maximum number of smORFs for identified lncRNAs with a six-frame translation mode. Furthermore, the implementation of multiple biological experiments further promoted the MS-based SEP identification. As shown in Figure 5D, the higher proportion of Lysine residues in SEPs suggests that single protease, such as trypsin digestion, may be not a good method for SEP identification, because it can produce very short peptides, which may not suitable for MS identification. On the contrary, multiple proteases may be a good choice to improve the SEP discovery.

LncRNAs have been considered as transcripts longer than 200 nt with little or no coding capacity, which is calculated using bioinformatic tools, including PLEK (Li et al., 2014), CPC (Kong et al., 2007) and CPAT (Wang et al., 2013). However, discovery for functional small peptides has prompted scientists to reconsider the definition and mechanism of lncRNAs. Some lncRNAs with small ORFs might encode a short peptide with important biological functions. MS data revealed that 115 herbivore-related lncRNAs encoding 302 SEPs. Furthermore, 61 SEPs were detected at least two time-points in *S. litura* attack samples, compared with control samples. Experimental evidences, including subcellular localization, MS, western plotting, and multiple bioinformatics analyses, including 3D structure prediction, confirmed the existence of several novel SEP-coding lncRNAs in tobacco. Commonly, subcellular localization is one of important factors for understanding the biological function of lncRNAs. In order to enable ribosomal translation, we expected more SEP-encoding lncRNAs would locate in the cytoplasm rather than nucleus. Bioinformatics results revealed that more SEP-encoding lncRNAs were predicted to locate in the cytoplasm (Figure 4A). However, as shown in Figures 4A,D, the subcellular location results were not very consistent between bioinformatics prediction tools and transient expression experiments, which implied current bioinformatics prediction tools need to be improved on their training model. In addition, proteins are found across cell membranes and organelles if they contain more positively charged aa and a hydrophobic region (Saghatelian and Couso, 2015). Results in Figure 5D also indicated that our identified SEPs trended to function as transmembrane peptides. Overall, both of them suggested these novel SEP-coding lncRNAs had the ability outside of the nucleus and potential ribosomal translation. Hence, we speculated that these herbivore attack response-related SEP-encoding lncRNAs might play important roles by small peptides. These potentially novel SEP-encoding lncRNAs might enable further investigation of the roles and mechanisms of lncRNA action in plants.

Most known functional small peptides in humans and mice are conserved between other species, implying that there might have some highly conversed short functional regions in lncRNAs. However, a search of our identified SEPs against the plant small-peptide database (PlantProDB; Das et al., 2020) revealed that none matched the existing sequences well. This may be because of the limited number of small peptides collected in PlantProDB, which contains only 6,087 small peptides for protein-encoding genes. In other words, studies of small peptides in plants are scarce. Compared to humans and other animals (Pauli et al., 2014; Matsumoto et al., 2017; Zhang et al., 2021), the coding nature and functional exploration of lncRNAs in plants is still almost unknown, and this research area remains a mystery. Hence, more related studies and databases for small peptides in plants are urgently required.

In summary, we have demonstrated that a comprehensive MS-based pipeline allows for the effective identification of large number of novel SEP-encoding lncRNAs in tobacco. Our study not only revealed potential lncRNA regulators of plant responses to herbivore attack, but also serves as a useful resource for the functional characterization of SEP-encoding lncRNAs.

## Data availability statement

The data presented in the study are deposited in BIG Data Center (http://bigd.big.ac.cn/gsa) repository, accession number: CRA007121 https://ngdc.cncb.ac.cn/gsa/browse/CRA007121.

## Author contributions

JJ and PC designed the experiments. JJ, YX, ZWL, ZFL, SY, and PL performed the bioinformatics analysis. LM, KC, XY, ZY, LC, and CW did the molecular biology experiment. JJ, JT, and PC wrote the manuscript. All authors approved the final version.

## Footnotes

1
http://pymol.org/

2
https://saves.mbi.ucla.edu/
