# Supplementary material for: Analysis of herbivore-responsive long noncoding ribonucleic acids reveals a subset of small peptide-coding transcripts in Nicotiana tabacum
Source: Front Plant Sci. 2022 Sep 23;13:971400. doi: 10.3389/fpls.2022.971400 (PMC9538394; doi:10.3389/fpls.2022.971400)
Supplement: Supplementary file 2 [file Data_Sheet_1.docx]

Supplementary Material

# Supplementary Data

The raw sequence data reported in this paper have been deposited in the Genome Sequence Archive (Chen et al., 2021) in National Genomics Data Center (Members and Partners, 2022), China National Center for Bioinformation / Beijing Institute of Genomics, Chinese Academy of Sciences (GSA: CRA007121) that are publicly accessible at https://ngdc.cncb.ac.cn/gsa.

# Supplementary Figures and Tables

## Supplementary Figures


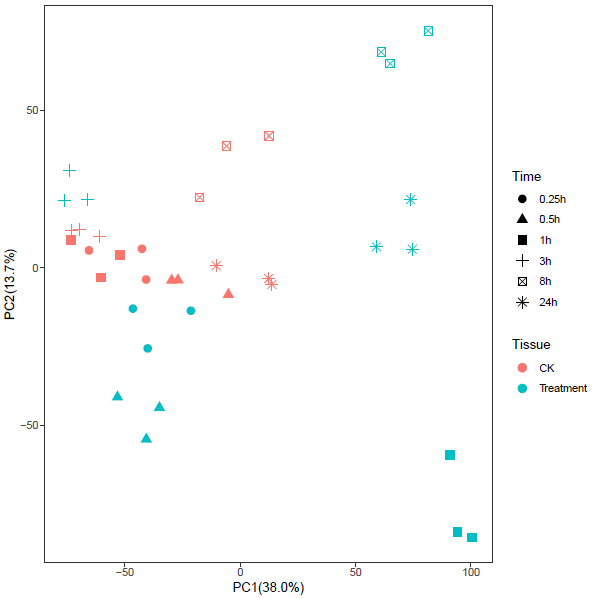


**Supplementary Figure 1.** PCA of genes from control and treated samples at each time-point.


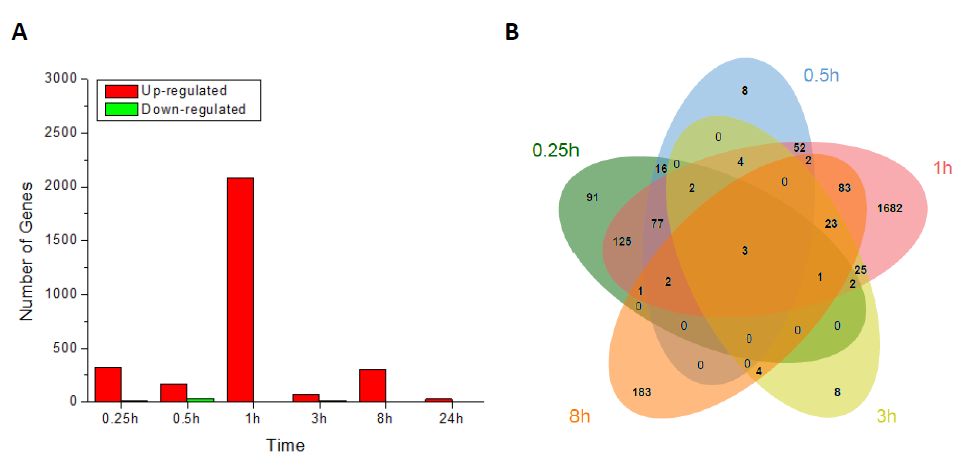


**Supplementary Figure 2.** The information for differentially expressed genes in *N. tabacum* after *S. litura* elicitation.

(A) Number of differentially expressed genes after *S. litura* elicitation.

(B) Venn graph between different time points for up-regulated genes after *S. litura* elicitation.


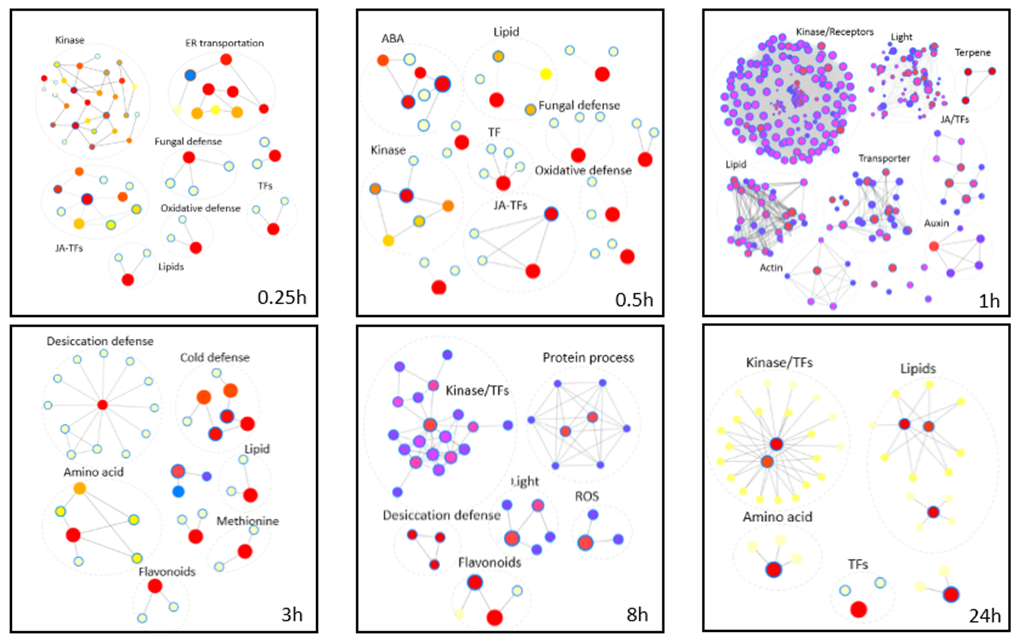


**Supplementary Figure 3.** Minimum connected molecular networks based on the up-regulated differential expressed genes in each time point.  **Note**: protein-protein interactions were retrieved from STRING databases. Ellipses mark groups of genes functionally related.


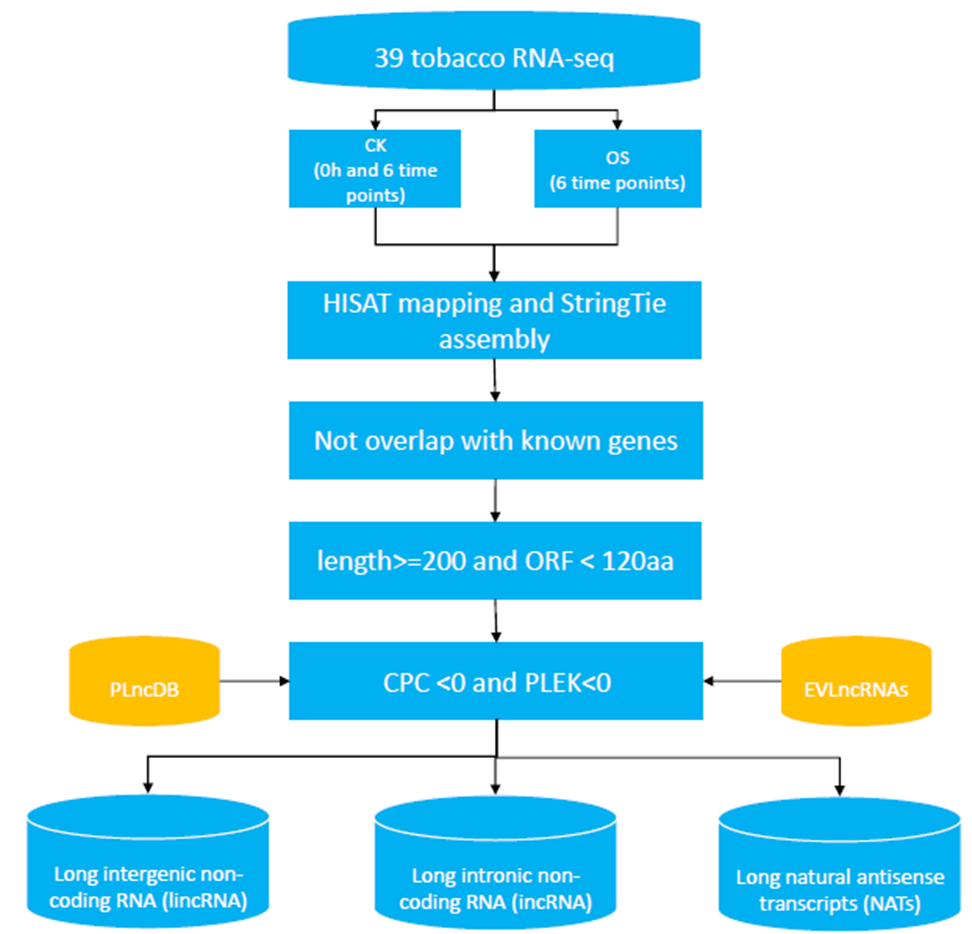


**Supplementary Figure 4.** The workflow of lncRNA identification.


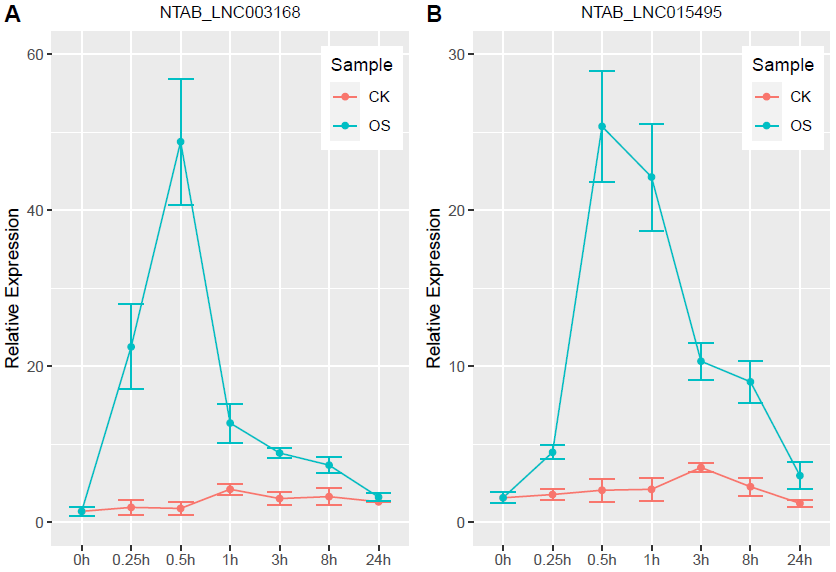


**Supplementary Figure 5.** qRT-PCR for lncRNAs NTAB_LNC003168 (A) and NTAB_LNC015495 (B). Error bars indicate SD (n = 3).


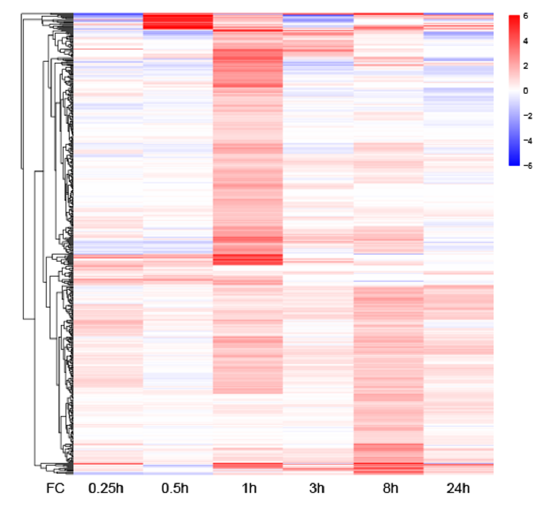


**Supplementary Figure 6.** Heatmap representing the transcript abundance FC of up-regulated lincRNAs in treated leaves compared with control leaves.


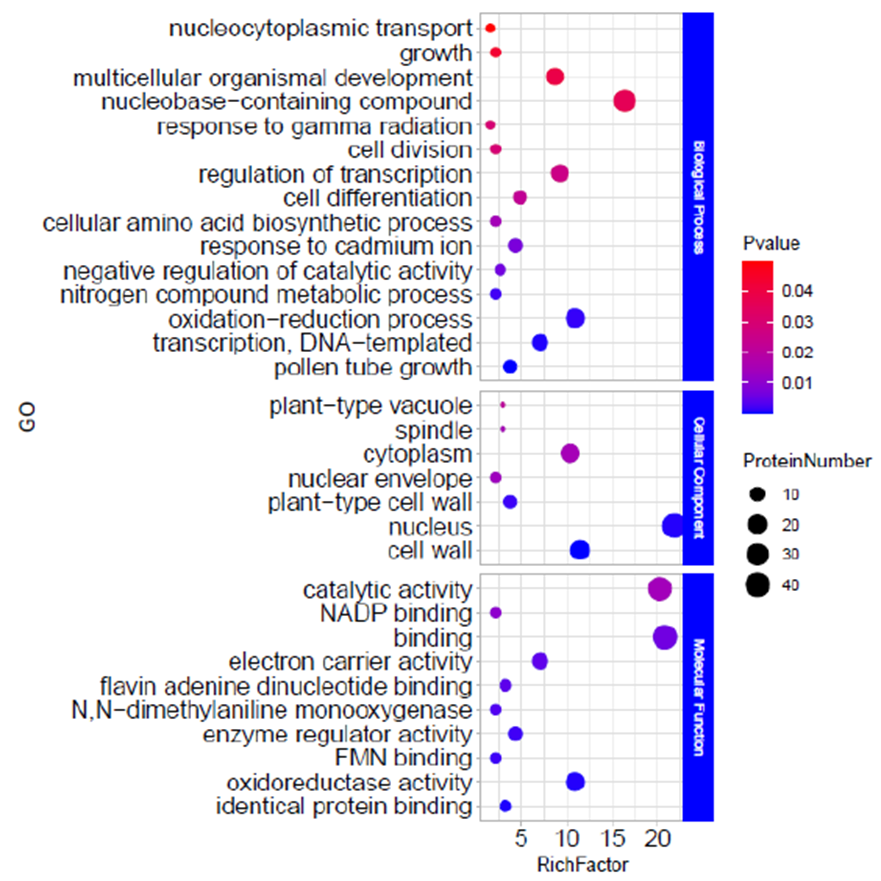


**Supplementary Figure 7.** GO enrichment analysis of neighboring genes around herbivore responsive lincRNAs.


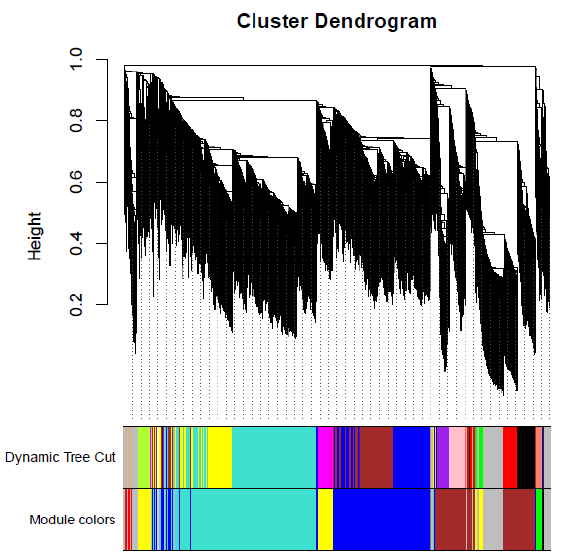


**Supplementary Figure 8.** The module information by WGCNA analysis based on differentially expressed genes and lncRNAs.


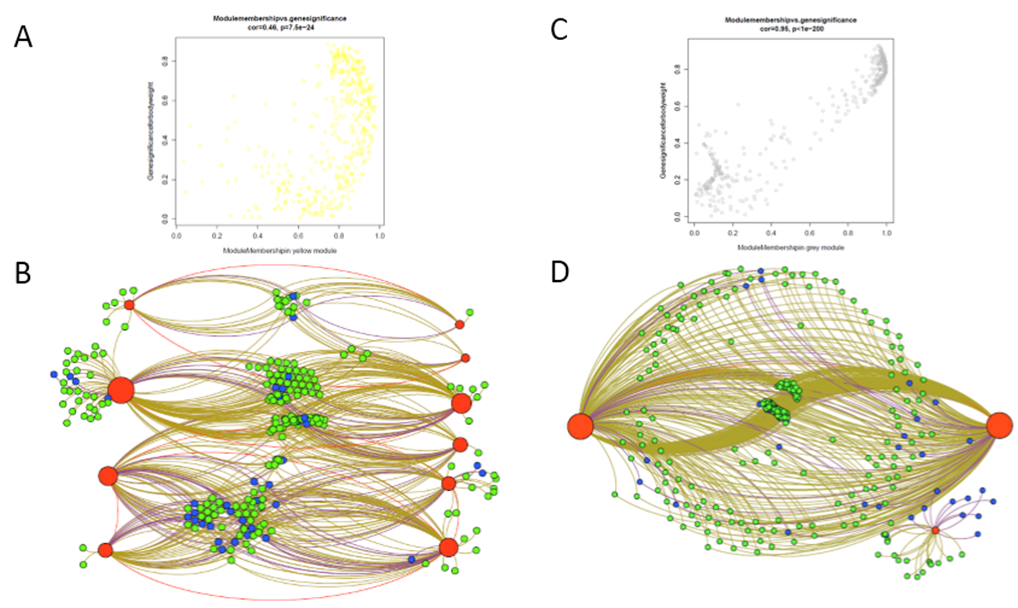


**Supplementary Figure 9.** Gene significance and network plot for yellow and grey modules.

(A) Gene significance plot between genes in yellow module and JA signal.

(B) Network plot between genes in JA biosynthesis pathways and other genes/lncRNAs in yellow module. Red color represents JA biosynthesis genes; Green color represents other protein-coding genes; Blue color represents lncRNAs.

(C) Gene significance plot between genes in grey module and JA signal.

(D) Network plot between genes in JA biosynthesis pathways and other genes/lncRNAs in grey module. Red color represents JA biosynthesis genes; Green color represents other protein-coding genes; Blue color represents lncRNAs.


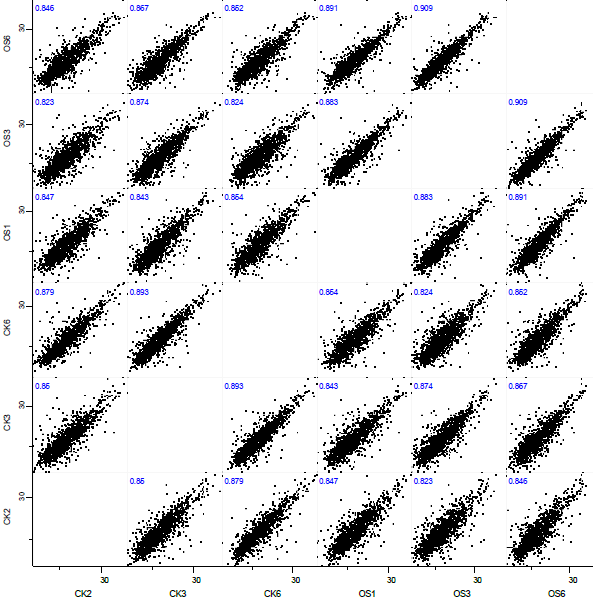


**Supplementary Figure 10.** Correlation between different biological replicates for each MS sample.


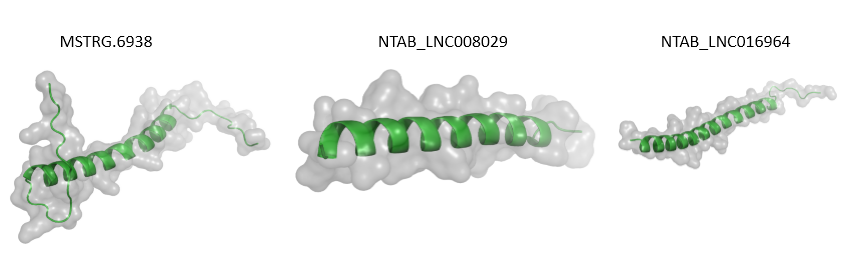


**Supplementary Figure 11.** Predicted 3D structures for selected SEPs.

.


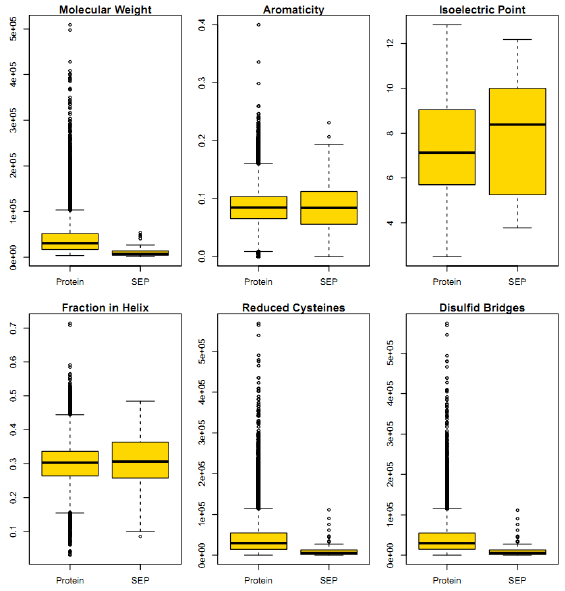


**Supplementary Figure 12.** Box plot for various protein features between canonical proteins and lncRNA-encoded SEPs, including molecular weight, aromaticity, isoelectric point, fraction in helix, reduced cysteines and disulfide bridges.

**Reference**

Chen, T., Chen, X., Zhang, S., Zhu, J., Tang, B., Wang, A., et al. (2021). The Genome Sequence Archive Family: Toward Explosive Data Growth and Diverse Data Types. *Genomics Proteomics Bioinformatics* 19(4)**,** 578-583. doi: 10.1016/j.gpb.2021.08.001.

Members, C.-N., and Partners (2022). Database Resources of the National Genomics Data Center, China National Center for Bioinformation in 2022. *Nucleic Acids Research* 50(D1)**,** D27-D38. doi: 10.1093/nar/gkab951.
