# Supplementary figures and images for: Analysis of herbivore-responsive long noncoding ribonucleic acids reveals a subset of small peptide-coding transcripts in Nicotiana tabacum
Source: Front Plant Sci. 2022 Sep 23;13:971400. doi: 10.3389/fpls.2022.971400 (PMC9538394; doi:10.3389/fpls.2022.971400)

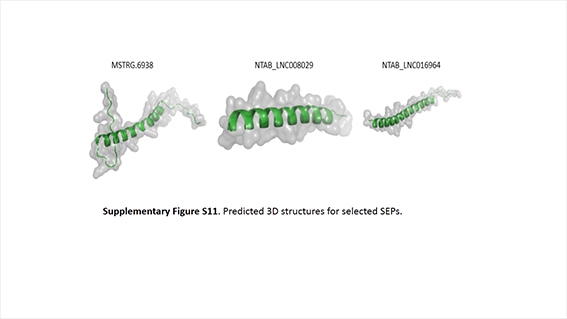

Supplement: Supplementary file 3 [file Image_1.TIF]

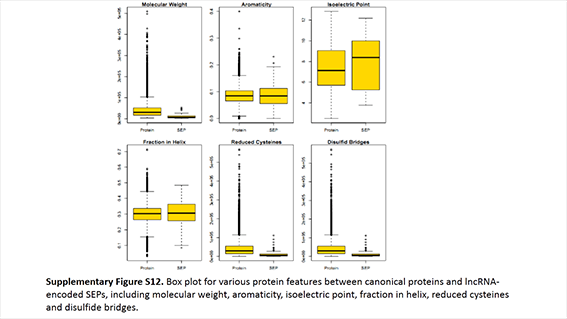

Supplement: Supplementary file 4 [file Image_2.TIF]
